# National research guideline for prehospital emergency medical care: A prospective Delphi-study

**DOI:** 10.15537/smj.2022.43.11.20220570

**Published:** 2022-11

**Authors:** Abdulrhman S. Alghamdi, Ahmed M. Alotaibi, Khaled J. Alshammari, Abdulrahman J. Alharbi, Rayyan S. Alrabiah, Abdulmohsen Y. Hadadi, Mohammed M. Albloushi, Abdullah A. Alabdali

**Affiliations:** *From the College of Applied Medical Sciences (Alghamdi, Alotaibi, Alshammari, Alharbi, Alrabiah, Hadadi, Albloushi, Alabdali), King Saud bin Abdulaziz University for Health Sciences; from King Abdullah International Medical Research Center (Alghamdi, Alotaibi, Alshammari, Alharbi, Alrabiah, Hadadi, Albloushi, Alabdali), Riyadh, Kingdom of Saudi Arabia; and from the Division of Cardiovascular Sciences (Alotaibi), University of Manchester, Manchester, United Kingdom.*

**Keywords:** emergency medical service, research, Saudi Arabia

## Abstract

**Objectives:**

To identify the most important research topics to establish a national research agenda and protocol for prehospital research in Saudi Arabia (KSA).

**Methods:**

A 3-round modified Delphi consensus methods were used to determine high-priority research topics. Round I included an open-ended question to list all high-priority research topics in a prehospital setting in Riyadh, KSA. Rounds II and III included ranking evaluation and consensus agreement. The included topics were listed based on the agreement of ≥70% of the experts participating in the study. The study was carried out between November 2021 and February 2022.

**Results:**

In total, 100 prehospital experts in KSA were invited to participate in all 3 rounds. Of these, 47 responded in round I, 34 in round II, and 39 in round III. In round I, participants submitted 278 research topics. After deduplication and sorting, 78 topics were assessed in the other 2 rounds.

**Conclusion:**

In this modified Delphi study, an expert panel identified the top prehospital emergency medical services (EMS) care research priorities. The leading research priorities included clinical and operational ideas. The proposed 32 high-priority topics can be used to guide researchers, research networks, policymakers, and funding organizations involved in EMS.

**T**he quality of prehospital emergency care for populations can be improved by using emergency medical services (EMS). A number of stakeholders, professionals, and researchers have created prehospital emergency care-related study agendas.^1-4^ Numerous recent studies have been carried out to examine the priorities for prehospital emergency care research, some of which have focused on specific aspects of prehospital treatment (such as disaster, pediatric, etc.).^1-4^

To efficiently target research that has the highest potential public health value, the process of establishing research priorities is crucial for researchers in the medical sector.^5,6^ While there are many ways to prioritize health research, there is no consensus on what constitutes the best approach. Setting health research priorities is essential to efforts to create national health research systems.^7^ Many researchers in different healthcare fields have adapted and utilized this approach.

Several worldwide studies have prioritized prehospital emergency care research. A research agenda is a significant part of research.^8^ The desirability approach in such a plan is to include a wide range of stakeholders and experts in a structured manner.^9-11^ Therefore, the main objective of this study was to develop and establish a prehospital emergency setting research guideline with the help of experts in the prehospital care settings in KSA. This will lead to an improvement in research quality in KSA, which, in turn, will enhance health care in the prehospital setting.

## Methods

A 3-round modified Delphi survey technique was used to identify research topic consensus among experts in prehospital care settings in Riyadh, KSA. Prehospital care experts were identified based on their involvement in prehospital policy, practice, and research. The modified Delphi methodology is an iterative process of multiple rounds of expert voting to reach a consensus in situations where there is minimal or no evidence and expert opinion is significant.^12^ The study was carried out between November 2021 and February 2022.

In the first round, research topics were generated. In the second round, the topics submitted from the first round were evaluated, and the presence or absence of consensus was determined. In the third round, we verified and validated if consensus existed or not. A predefined consensus threshold was set at ≥70% in both the second and third rounds for either inclusion or exclusion.

The study team made use of their prior expertise to approach possible policymakers, specialists, and researchers in prehospital emergency care. The team agreed to nominate participants from different specialties working in various Saudi Arabian organizations. The eligibility criteria were as follows: I) academicians or emergency clinicians with expertise in emergency medicine or prehospital emergency care; II) researchers and experts with work experience of at least 3 years. Participants who did not meet the inclusion criteria were excluded from the study.

Prior to the study, an Institutional Review Board approval was obtained from King Abdullah International Medical Research Center, Riyadh, KSA (IRBC/1971/21).

The study team identified and invited 100 participants for the 3 rounds. The identified experts were: I) emergency medicine physicians; II) academicians or researchers in prehospital emergency care; III) paramedics; and IV) emergency nurses. Additionally, all experts involved in this study had at least 3 years of experience in either prehospital emergency research or care. Thus, we expected a variation in opinions among the participants based on their clinical experience, research experience, interest, education level, and career level. Finally, no sample size calculation was carried out as Delphi studies rely on reaching a consensus rather than a sample size.^13^ In addition, all 100 participants were contacted in all 3 rounds.

### Statistical analysis

After collating all responses from round I, 2 investigators merged the suggestions that noticeably indicated the same research topic and excluded the responses that were undoubtedly irrelevant to the prehospital setting. In rounds II and III, for each research topic, 3-point Likert scale responses were presented using frequencies and percentages.

The threshold for inclusion consensus was identified as ≥70% of participants agreeing on a research topic, while the threshold for exclusion consensus was identified as ≥70% of participants disagreeing on a topic. Microsoft 365 Excel for Mac, version 16.65 (Microsoft, Redmond, WA., USA) was carried out for data analysis.

## Results

In round I, 47 (47%) of 100 participants invited through email completed the online survey. Most round I respondents (n=22, 46.8%) were paramedics. Furthermore, most participants (n=15, 31.9%) had 3-5 and 11-15 years of experience (Table 1). In round II, 34 (34%) of the selected participants completed the online survey. Like round I, most of these responders were paramedics (n=20, 58.8%), and most participants had at least 3-5 years of experience (n=13, 38.2%). In round III, 39 (39%) of all selected participants completed the online survey. Most of these responders were paramedics (n=18,46.2%) and most participants had 11-15 years of experience (n=13, 33.3%; Table 1).

**Table 1:** - Participants’ demographics.

Variables	Round I	Round II	Round III
Role	47	34	39
Emergency medicine physician	9 (19.1)	2 (5.9)	7 (17.9)
Paramedic	22 (46.8)	20 (58.8)	18 (46.2)
Pre-hospital academic or researcher	14 (29.8)	11 (32.4)	12 (30.8)
Emergency nurse	2 (4.3)	1 (2.9)	2 (5.1)
***Years of experience***			
3-5	15 (31.9)	13 (38.2)	11 (28.2)
6-10	11 (23.4)	9 (26.5)	9 (23.1)
11-15	15 (31.9)	8 (23.5)	13 (33.3)
16-20	4 (8.5)	4 (11.8)	6 (15.4)
>21	2 (4.3)	0 (0.0)	0 (0.0)

Values are presented as a number and percentage (%).

In round I, participants were requested to list all the research topics they felt required investigation. A total of 47 participants provided 287 statements. After deduplication, sorting, and removing irrelevant statements, 78 research topics were eligible for inclusion in round II.

**Figure 1:**
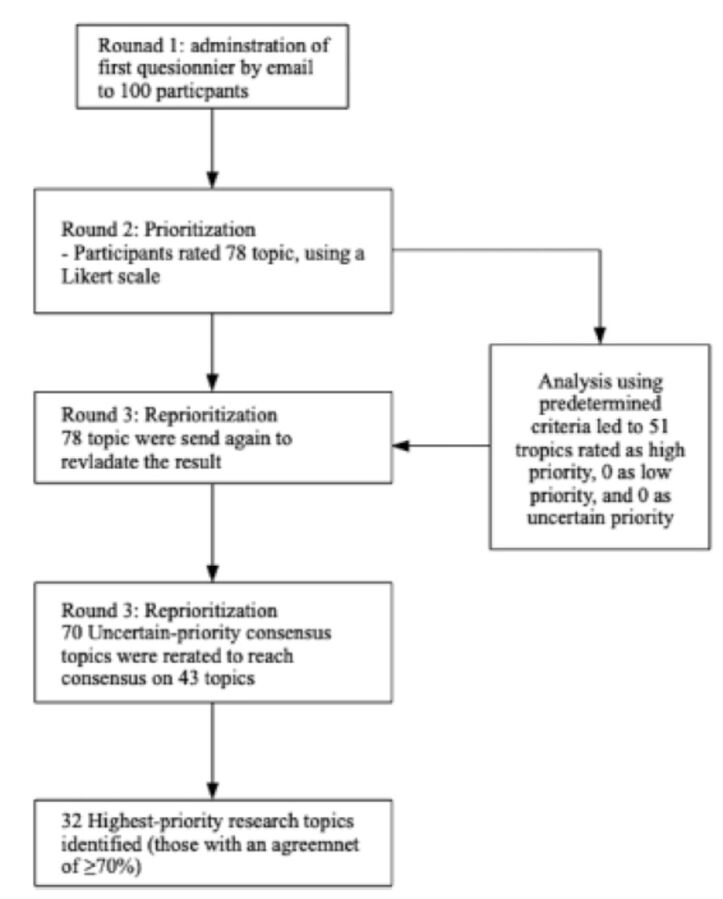
- Flow chart of the study process.

In round II, the participants were requested to categorize the 78 topics on a 3-point Likert scale. The results were sorted into high, low, and uncertain priority categories, in which 51 topics were included as they met the inclusion threshold, and none met the threshold for exclusion. The remaining 26 topics met the non-consensus threshold. Moreover, round III included high and uncertain priority (non-consensus) topics for further assessment. Therefore, all 78 topics were included in the final round (round III). In round III, participants were again given the 78 topics from round II (Figure 1).

In round III, the participants were requested to categorize all 78 topics using a 3-point Likert scale. This resulted in 32 topics meeting the inclusion threshold (Table 2). Additionally, the experts changed their opinion regarding 20 topics that had met the inclusion consensus in round II to non-consensus in this round (Table 3).

**Table 2:** - Research priorities met, including criteria from round II and III.

No.	Suggestions	Round II agreement (%)	Round III agreement (%)
1	Reliability of emergency medical services records	97.1	89.7
2	Pediatric care	82.4	89.7
3	Epidemiology of out-of-hospital cardiac arrest	85.3	87.2
4	Level of crew training impact on patient outcome	91.2	84.6
5	Effectiveness of prehospital critical care	88.2	84.6
6	Dispatch pre-arrival assistance	85.3	84.6
7	Prehospital pain management	82.4	84.6
8	Clinical protocols and guidelines improvement and implementation	91.2	82.1
9	Survival rate following out-of-hospital cardiac arrest	82.4	82.1
10	Emergency medical services response to stroke	82.4	82.1
11	Integration and telecommunication between prehospital systems and hospitals	91.2	79.5
12	The role of home health care in reducing emergency medical services calls	88.2	79.5
13	Prehospital response and scene time	85.3	79.5
14	The efficiency of prehospital systems	82.4	79.5
15	The outcome of dispatcher-assisted cardiopulmonary resuscitation	79.4	79.5
16	Challenges in providing care for special population groups (bariatric, geriatrics, pediatric, and mental health patients)	88.2	76.9
17	Obstetrics and gynecologyand neonatal care in prehospital	85.3	76.9
18	Awareness for the public on when to call for an ambulance	82.4	76.9
19	Workforce psychological stress and anxiety	79.4	76.9
20	Impact of COVID-19 pandemic on emergency medical services	76.5	76.9
21	Cardiopulmonary resuscitation quality	70.6	76.9
22	Continuous education and training	82.4	74.4
23	Prehospital safety measures	82.4	74.4
24	Time of response and transportation of critically ill patients	79.4	74.4
25	Epidemiology of traumatic brain injury	79.4	74.4
26	Role and importance of air ambulance in transportation	79.4	74.4
27	Accessibility to an automated external defibrillator	76.5	74.4
28	Occupational burnout	76.5	74.4
29	Trauma registry	82.4	71.8
30	Physical fitness among prehospital care providers	82.4	71.8
31	Infection in prehospital settings	79.4	71.8
32	Patient’s safety	76.5	71.8

**Table 3:** - Research priorities excluded after round III.

No.	Suggestions	Round II agreement (%)	Round III agreement (%)
1	Occupational health and risk of physical injuries prevention	82.4	69.2
2	Dispatch role in crises	76.5	69.2
3	Readiness and training level among emergency medical services specialists to respond to mass casualty incidents	73.5	69.2
4	Post-traumatic stress disorder	73.5	69.2
5	Innovations in emergency medical services curriculum: what did we learn from the pandemic?	73.5	69.2
6	Emergency medical services simulation integrated curriculum	82.4	66.7
7	Workforce anxiety	76.5	66.7
8	The impact of new paramedic decisions on patients’ safety	76.5	66.7
9	Fluid resuscitation in pre-hospital trauma patients	73.5	66.7
10	Barriers and consequences of delaying delivery	82.4	64.1
11	Identification and management of sepsis	79.4	64.1
12	Preparedness of emergency medical services to deal with multi-casualty accidents	79.4	64.1
13	The access, quality, and costs of pre-hospital care	73.5	64.1
14	Workforce mental health and wellbeing	73.5	64.1
15	Successful intubation and patient outcome	70.6	64.1
16	Epidemiology of myocardial infarction	76.5	59.0
17	Helicopter emergency medical services utilization	76.5	56.4
18	Emergency medical services role in hospital-based emergency medical services stations	70.6	56.4
19	Workforce sleep disorder	73.5	51.3

## Discussion

This study carried out a gap analysis for prehospital research by inviting clinicians, researchers, and experts interested in prehospital emergency care to initiate and determine the top research priorities for prehospital emergency care. A total of 32 research topics met the inclusion threshold (≥70% agreement) and were considered important.

Several studies have reported the importance of repeating rounds for consensus studies, allowing participants to reflect on alternate views they may have missed and reconsider their initial responses.^14,15^ In our study, the significance of repeating the ranking evaluation in round III for all topics that reached the inclusion consensus increased its validity. By doing so, the experts re-ranked all 51 included topics from round II. Only 32 out of 51 topics reached the inclusion consensus. As a result, only high-priority topics were agreed upon by the experts.

The ideas developed and prioritized in this study represent a comprehensive list generated by prehospital emergency care stakeholders and experts from various backgrounds and experiences using the Delphi methodology. The results of this study are intended to serve as a guideline for future prehospital research and related funding.

Patient care and outcomes were essential components of the high-priority topics in our study. Both were mentioned in the context of observational and interventional research (namely, the effectiveness of prehospital critical care, survival rate following out-of-hospital cardiac arrest, and prehospital pain management). In addition, experts provided several ideas related to system-level research and system benchmarks. Despite the importance of system benchmarks in the process, time, and efficiency of care, the main emphasis of experts’ view was based on ideas related to improving patient’s care and reducing mortality. This reflects the significant effort the study participants placed on designing prehospital research studies to impact patient’s outcomes and save lives. Likewise, several previous health care research priority studies focused on patient care outcomes.^16-20^ Similarly, international studies have explored prehospital emergency care focusing on patient care outcomes.^1-4^

### Study limitations

The response rate in round II was relatively low (32%). However, the Delphi study aims to have a good representation of research ideas from the participants rather than getting a high response rate, which was achieved. Despite the extensive list of research topics, the study focus was to determine prehospital research priorities in KSA, so the results of this Delphi study may not apply to other counties.

In conclusion, in this modified Delphi study, an expert panel identified the top prehospital EMS care research priorities. The leading research priorities included clinical and operational ideas. The proposed 32 high-priority ideas can be used to guide researchers, research networks, policymakers, and funding organizations involved in EMS. The study’s implication is to determine research priorities which will be provided to the Saudi Research, Development and Innovation Authority, Ministry of Health, Saudi Red Crescent Authority, and research centers to disseminate our results, apprise future prehospital research plans, and prioritize funding. Overall, the results of this study can significantly improve the care and quality of EMS.
